# Risky behaviours among young people living with HIV attending care and treatment clinics in Dar Es Salaam, Tanzania: implications for prevention with a positive approach

**DOI:** 10.7448/IAS.16.1.17342

**Published:** 2013-10-11

**Authors:** Aisa Mhalu, Germana H Leyna, Elia J Mmbaga

**Affiliations:** 1 Management and Development for Health, Dar Es Salaam, Tanzania; 2 Department of Epidemiology and Biostatistics, Muhimbili University of Health and Allied Sciences, Dar Es Salaam, Tanzania

**Keywords:** HIV, young people, risky behaviours, Tanzania

## Abstract

**Introduction:**

Prevention with a positive approach has been advocated as one of the main strategies to reduce new instances of HIV infection. Risky sexual behaviours among people living with HIV/AIDS are the cornerstone for this approach. Understanding the extent to which infected individuals practice risky behaviours is fundamental in designing appropriate population-specific interventions. With the HIV infection transmission rates remaining high among young people in sub-Saharan Africa, continued prevention among them remains a priority. This study therefore seeks to describe the magnitude and determinants of risky sexual behaviours among young people living with HIV.

**Methods:**

A cross-sectional study was conducted between June and July 2010 in selected Care and Treatment Clinics (CTCs) in Dar Es Salaam, Tanzania. A total of 282 HIV-positive patients aged 15–24 were interviewed about their sexual behaviours using a questionnaire.

**Results:**

Prevalence of unprotected sex was 40.0% among young males and 37.5% among young females (*p*<0.001). Multiple sexual partnerships were reported by 10.6% of males and 15.9% of females (*p*<0.005). More than 50% of the participants did not know about the HIV status of their sexual partners. A large proportion of participants had minimal knowledge of transmission (46.7% males vs. 60.4% females) and prevention (65.3% males vs. 73.4% females) of sexually transmitted infections (STIs). Independent predictors of condom use included non-use of alcohol [adjusted odds ratio (AOR), 0.40 95% confidence interval (CI); 0.17–0.84] and younger age (15–19 years) (AOR, 2.76, 95% CI: 1.05–7.27). Being on antiretroviral therapy (AOR, 0.38, 95% CI: 0.17–0.85) and not knowing partners’ HIV sero-status (AOR, 2.62, 95% CI: 1.14–5.10) predicted the practice of multiple sexual partnership.

**Conclusions:**

Unprotected sex and multiple sexual partnerships were prevalent among young people living with HIV. Less knowledge on STI and lack of HIV disclosure increased the vulnerability and risk for HIV transmission among young people. Specific intervention measures addressing alcohol consumption, risky sexual behaviours, and STI transmission and prevention knowledge should be integrated in the routine HIV/AIDS care and treatment offered to this age group.

## Introduction

Out of the 1.7 billion young people worldwide, it is estimated that 5.4 million of those aged 15–24 are living with HIV/AIDS, and 6000 are being infected daily. Sub-Saharan Africa is home to 63% of young people living with HIV/AIDS [1]. Despite infected individuals either abstaining or reducing sexual activity, about 10–64% continue to engage in risky sexual behaviours [2]. Young people infected with HIV have been reported to have a higher prevalence of risky sexual behaviours than the general population even after diagnosis [3, 4]. It has been estimated that at least one-third of HIV-positive young people may maintain their risk behaviours even after learning their sero-status [5–7]. Young people living with HIV do perceive themselves as being victims of their own behaviours and thus may not see the need to protect themselves from HIV and other sexually transmitted infections (STIs) [8, 9]. In addition, the very psychosocial and socio-economic factors that place young people at risk for acquiring HIV are the same ones that leave them at the margins of disease prevention and healthcare systems [10].

The prevalence of HIV infection among young people aged 20–24 in Tanzania is estimated to be 3.2% (4.4% for females and 1.7% for males) [11]. Recent studies indicate the age of first sexual encounter for young people aged 16–19 to be 14 years with an incidence of first sexual encounter of 17 per 100 person-years at risk [12]. The same study revealed that almost half of the young people engaged in multiple sexual partnerships with about 40% of young females exchanging sex for money. Furthermore, a multi-country intervention study involving Tanzania indicated the rate of unprotected sex to be high with only 20% of young people aged 12–14 reporting to have used a condom during their last sexual encounter [13]. Young people living with HIV may continue to engage in sexual behaviours that place their partners at risk of HIV infection [14]. The practice of risky sexual behaviours among HIV-positive individuals is the most effective driver of the HIV epidemic. This has recently called for the prevention with a positive approach of HIV transmission. Positive prevention approach focuses on the notion that appropriate treatment reduces viral load and hence reduces transmission probability. Moreover, this approach takes into consideration behavioural factors that may result in increased HIV transmission to uninfected sexual partners. Several studies focusing on the general population have examined the magnitude of risky sexual behaviours in Tanzania but no study has specifically examined these behaviours among young people living with HIV. Understanding the magnitude and predictors of such behaviours among this group remains a priority as this information will inform efforts to include prevention with positive messages in the routine HIV/AIDS care and treatment of young people. Therefore, this study intends to describe the prevalence and predictors of HIV transmission-related behaviours of young people living with HIV/AIDS in an urban setting of Tanzania.

## Materials and methods

### Study design and sampling

A cross-sectional study of 15- to 24-year-old HIV-positive patients receiving care and treatment at eight care and treatment clinics (CTCs) in Temeke, Ilala and Kinondoni municipalities of Dar Es Salaam, Tanzania, was conducted between June and July 2010. The clinics provide antiretroviral therapy (ART) and counselling regarding drug adherence and reproductive health. A multi-stage random sampling method was employed to select eight health facilities/clinics from the city, where the number of health facilities/clinics selected from each municipality was proportional to the number of facilities in each municipality. Lists of all eligible young people receiving care at the selected health facilities/clinics were obtained and systematic random sampling was done. Eligibility was based on being infected with HIV regardless of the mode of transmission, age and having received care at the clinic for a period of six months or more. Participants were recruited during their routine scheduled clinic days.

### Data collection methods

A questionnaire was used to collect data through face-to-face interviews. Data on socio-demographic characteristics, sexual behaviours, knowledge regarding HIV and STI transmission, symptoms, complication and prevention; psychosocial factors such as family support and perceived health status were collected from all participants. Information on the use of ART was obtained from patient files.

### Data analysis

Categorical data were summarized using frequencies and differences between proportions assessed using χ^2^ test. Continuous variables were summarized by calculating the mean and standard deviation and student's *t*-test was used to test statistical differences between means. HIV/STI knowledge scales were created with a score that ranged from 0 to 10. Young people scoring 8 and above were considered to have high knowledge, while those scoring 5–7 had moderate knowledge and those scoring 0–4 were considered to have minimal knowledge. Having more than one lifetime sexual partner was categorized as having multiple sexual partners.

Backward stepwise logistic regression models were built to identify independent predictors of risky sexual behaviours. All predictors with a *p*-value of 0.2 and below from bivariate analysis were entered into the full multi-variable logistic regression model. Risk-sexual behaviour variables with the potential for multi-collinearity were examined based on a correlation matrix and checked again by the use of variance inflation factor. Variance infection factor of 10 was considered as a diagnostic value as suggested by Myers [15]. Adjusted odds ratio and 95% confidence intervals are presented. Significance level was set at 0.05 and all of the analyses were two-tailed. Data were analyzed using SPSS version 15 programme.

### Ethical consideration

Muhimbili University of Health and Allied Sciences ethical committee approved the study protocol. Permission was also sought from participating health facilities to conduct the study on-site. Parents of adolescents aged less than 18 gave written consent and their children gave agreement to participate in the study. Participants aged 18 or older gave written consent. All of the interviews were conducted in a private place and participant's confidentiality was maintained throughout the study.

## Results

A total of 282 HIV-positive patients aged 15–24 attending CTC in Dar Es Salaam were recruited in the study, of whom 207 (73.4%) were female. The mean age of the respondents was 20.5 (SD=3.1) years and did not differ between males and females. Almost half of the respondents reported to have completed primary education and the distribution of education status of the respondents did not significantly differ between males and females (*p*=0.914). The majority of the respondents in this study were single, particularly male respondents and a significantly large proportion of females were married as compared to their male counterparts (*p*<0.001). The social–demographic characteristics of the respondents by sex are shown in Table 1.

**Table 1:** Distribution of social–demographic characteristics of respondents by sex in Dar Es Salaam, Tanzania

Characteristics Sex	Male (N=75) n (%)	Female (N=207) n (%)	χ^2^ value	*p*
Age group (years)				
15–19	27 (36.0)	83 (40.0)	0.388	0.582
20–24	48 (64.0)	124 (60.0)		
Educational level				
No formal education	5 (6.6)	12 (5.8)	0.522	0.914
Primary education	38 (50.6)	101 (48.7)		
Secondary education	30 (40.0)	85 (41.0)		
University/college	2 (2.8)	9 (4.5)		
Religion				
Muslim	39 (52.0)	105 (50.7)	0.356	0.893
Christian	36 (48.0)	102 (49.3)		
Place of residence				
Temeke	21 (28.0)	60 (28.9)	0.409	0.815
Ilala	35 (46.6)	102 (49.3)		
Kinondoni	19 (25.4)	45 (21.8)		
Marital status				
Married	10 (13.3)	35 (16.9)	0.963	0.810
Single	54 (72.0)	139 (67.1)		
Cohabiting	6 (8.0)	21 (10.1)		
Divorced/separated/widowed	5 (6.7)	12 (5.9)		
Occupation				
Private business	14 (18.7)	50 (24.1)	1.965	0.854
Casual labour	6 (8.0)	12 (5.8)		
Student	23 (30.6)	66 (31.9)		
Housewife	13 (17.3)	34 (16.4)		
Employed	4 (5.4)	13 (6.3)		
Others	15 (20.0)	32 (15.5)		

### Magnitude of risky sexual behaviours

Table 2 depicts risky sexual behaviours among the sample of HIV-positive young people in Dar Es Salaam. About 15.9% of female and 10.6% of male respondents reported to have multiple sexual partners (*p*<0.001). Males were more likely to have their sexual debut at an age less than 15 years (84.8%) as compared to females (68.0%) (*p*=0.048). Female respondents were significantly more likely to report involvement in vaginal (*p*<0.001) and oral (*p*=0.019) sex than males.

**Table 2:** Practice of risky sexual behaviours among young people living with HIV who reported to have ever had sex in Dar Es Salaam, Tanzania

Risky behaviours		Male (N=75) n (%)	Female (N=207) n (%)	χ^2^ value	*p*

Variables	Category				
Unprotected sex on last sexual intercourse†	Yes	14 (40.0)	63 (37.5)	0.076	0.782
	No	21 (60.0)	105 (62.5)		
Number of sexual partners	0–1	67 (89.4)	174 (84.1)	4.132	0.002
	≥2	8 (10.6)	33 (15.9)		
Age of sexual debut‡	<15 years	28 (84.8)	116 (68.0)	3.865	0.048
	≥15 years	5 (15.2)	55 (32.0)		
Vaginal	Yes	34 (45.3)	169 (81.6)	35.991	0.000
	No	41 (54.7)	38 (18.4)		
Anal	Yes	4 (5.3)	14 (6.8)	0.188	0.664
	No	71 (94.7)	193 (93.2)		
Oral	Yes	19 (25.3)	84 (40.6)	5.519	0.019
	No	56 (74.7)	123 (59.4)		

† Include those who reported to have had sex during the last three months;

‡ include those who reported to ever had sex.

### Knowledge on STI

The level of knowledge on STI prevention was low with 60.4% of females and 46.7% of males categorized as having a minimal knowledge level. This difference between sexes had a borderline significance (*p*=0.073).

A substantial proportion of male (65.3%) and female (73.4%) respondents had less knowledge on STI transmission. However, knowledge on STI symptoms was relatively high (92% for males and 92.7% for females) with genital ulcers (50.4%) and genital itching (38.7%) being the symptoms most mentioned.

Knowledge about STI complications was relatively high among both male (65.3%) and female (74.9%) respondents. About half of the respondents (male 56% and female 52.2%) had moderate knowledge on types of STIs. Syphilis (247; 87.6%), gonorrhoea (237; 84%) and HIV infection (148; 52.5%) were most mentioned while Chlamydia, vaginal candidiasis and genital warts the least mentioned. Generally, STI knowledge did not differ between males and females in this population (*p*=0.073) (Table 3).

**Table 3:** Levels of different categories of STI knowledge among young people living with HIV in Dar Es Salaam, Tanzania

Knowledge levels	Male (N=75) n (%)	Female (N=207) n (%)	χ^2^ value	*p*
Knowledge on STI prevention				
Low	35 (46.7)	125 (60.4)	5.222	0.073
Moderate	32 (42.7)	71 (34.3)		
High	8 (10.6)	11 (5.3)		
Knowledge on STIs transmission				
Low	49 (65.3)	152 (73.4)	2.553	0.279
Moderate	22 (29.4)	50 (24.2)		
High	4 (5.3)	5 (2.4)		
Knowledge of STI symptoms				
Low	6 (8.0)	15 (7.2)	0.269	0.874
Moderate	34 (45.3)	101 (48.8)		
High	35 (46.7)	91 (44.0)		
Knowledge of STI complication				
Low	4 (5.3)	7 (3.4)	2.567	0.277
Moderate	22 (29.4)	45 (21.7)		
High	49 (65.3)	155 (74.9)		
Knowledge on types of STIs				
Low	2 (2.7)	8 (3.9)	0.462	0.794
Moderate	42 (56.0)	108 (52.1)		
High	31 (41.3)	91 (44.0)		

### Family support and practice of risky sexual behaviours

The relationship between family support and risky sexual behaviours among people living with HIV is summarized in Table 4. The majority (87.4%) of the respondents with family support reported having 0–1 sexual partners as compared to those without family support (75.0%) (*p*=0.018). A significantly lower proportion of young people with family support reported not having had sex since their HIV diagnosis as compared to those without family support (*p*=0.023).

**Table 4:** Relationship between young people's family support status and practice of risky behaviours among young people living with HIV in Dar Es Salaam, Tanzania

	How supportive are people in your family about your HIV status			
			
Risky sexual behaviours	Supportive n (%)	Not supportive n (%)	χ^2^ value	*p*
Use of condom for each sexual act				
Yes	99 (58.6)	15 (60.0)	0.180	0.893
No	70 (41.4)	10 (40.0)		
Number of sexual partners				
0–1	194 (87.4)	45 (75.0)	5.608	0.018
> 2	28 (12.6)	15 (25.0)		
Alcohol use after HIV diagnosis				
Yes	49 (22.0)	8 (29.6)	0.778	0.377
No	173 (78.0)	19 (70.4)		
Substance use				
Yes	5 (2.3)	1 (3.7)	0.215	0.676
No	217 (97.7)	26 (96.3)		
Ever had sex since HIV diagnosis				
Yes	131 (59.0)	22 (81.5)	5.132	0.023
No	91 (41.0)	5 (18.5)		

### Use of antiretroviral and practice of risky sexual behaviours

Respondents who were on antiretroviral drugs were significantly more likely to report using a condom during their last sexual intercourse (66.7% vs. 55.4%), less engagement in multiple sexual partnerships (65.8% vs. 79.8%), less use of alcohol (20.7% vs. 31.5%), less practice of vagina sex (61.1% vs. 95.5%) and oral sex (29.5% vs. 51.7%) as compared to those who were not on ART, respectively (Table 5).

**Table 5:** Relationship between the use of antiretroviral therapy and practice of risky behaviours among young people living with HIV in Dar Es Salaam, Tanzania

Risk behaviours	On ART's, n (%)	Not on ART's, n (%)	χ^2^ value	*p*
Condom use last sexual intercourse				
Yes	80 (66.7)	46 (55.4)	7.124	0.000
No	40 (33.3)	37 (44.6)		
Substance use				
Yes	6 (3.1)	3 (3.4)	0.014	0.907
No	187 (96.9)	86 (96.6)		
Multiple sexual partnerships				
Yes	127 (65.8)	71 (79.8)	5.685	0.017
No	66 (34.2)	18 (21.2)		
Alcohol use				
Yes	40 (20.7)	28 (31.5)	3.846	0.050
No	153 (79.3)	61 (68.5)		
Vagina sex				
Yes	118 (61.1)	85 (95.5)	35.671	0.000
No	75 (38.9)	4 (4.5)		
Anal sex				
Yes	13 (6.7)	5 (5.6)	0.127	0.721
No	180 (93.3)	84 (94.4)		
Oral sex				
Yes	57 (29.5)	46 (51.7)	12.892	0.000
No	136 (70.5)	43 (48.3)		

### Independent determinants of unprotected sex and multiple sexual partnership

Participants from Kinondoni municipality were 62% less likely to report multiple sexual partnerships as compared to those from Temeke municipality (*p*=0.051).

Moreover, using ART was associated with a 62% lower likelihood of reporting engaging in multiple sexual partnerships as compared to those who were not on ART.

The likelihood of engaging in a multiple sexual partnership was 2.6 times higher among respondents who did not know about the HIV status of their sexual partners as compared to those who did (*p*=0.023) (Table 6).

**Table 6:** Logistic regression of the determinants of multiple sexual partnerships and unprotected sex during last sexual act among young people living with HIV in Dar Es Salaam, Tanzania

	Multiple sexual partnership		Unprotected sex	
		
	AOR (95% CI)*	*p*	AOR (95% CI)*	*p*
Age group				
20–24	1	0.705	1	0.040
15–19	1.23 (0.41–3.70)		2.76 (1.05–7.27)	
Age of sexual debut				
≤15	1	0.443	1	0.274
≥16	0.73 (0.33–1.63)		1.84 (0.62–5.46)	
Sex				
Male	1	0.884	1	0.613
Female	1.06 (0.49–2.29)		0.83 (0.35–1.96)	
Educational status				
No formal education	1		1	
Primary education	0.97 (0.29–3.17)	0.953	2.81 (0.50–15.72)	0.240
Secondary education	1.87 (0.42–5.52)	0.515	3.15 (0.49–20.46)	0.230
University/college	0.57 (0.08–3.97)	0.571	–	–
District of residence				
Temeke	1		1	
Kinondoni	0.38 (0.14–1.00)	0.051	1.00 (0.46–2.19)	0.993
Ilala	1.07 (0.30–3.90)	0.913	0.65 (0.23–1.79)	0.400
Knowledge on STI prevention				
Knowledgeable	1	0.483	1	0.188
Not knowledgeable	1.38 (0.560–3.41)		0.44 (0.13–1.40)	
Family support				
Supportive	1	0.943	1	0.844
Not supportive	1.04 (0.39–2.75)		1.13 (0.35–3.64)	
ART status				
Not on ART's	1	0.018	1	0.583
On ART's	0.38 (0.17–0.85)		1.25 (0.56–2.81)	
Knowing partner HIV status				
Yes	1	0.023	1	0.769
No	2.62 (1.14–5.10)		0.9 (0.31–1.24)	
Alcohol use				
Yes	1	0.473	1	0.017
No	1.1 (0.83–6.30)		0.40 (0.17–0.84)	

* Adjusted for all variables in the model. AOR, adjusted odds ratio; CI, confidence interval; STI, sexually transmitted infection; ART, antiretroviral therapy.

Respondents who were aged 15–19 were almost three times more likely to report engaging in unprotected sex as compared to their older counterparts (20–24 years) (*p*=0.040). Again, young people who reported not using alcohol were 60% less likely to report practicing unprotected sex as compared to those who were consuming alcohol (*p*=0.017) (Table 6).

## Discussion

Engaging in unprotected sex was found to be high in this sample of young people living with HIV. Moreover, a modest proportion of young people reported to have engaged in multiple sexual partnerships. Other studies in Tanzania and elsewhere have also reported higher risky behaviours among young people. Rongkavilit *et al*. reported inconsistent use of condoms among young people in Thailand to be as high as 55.6%. Tanzania Health and Malaria Indicator Survey of 2011/2012 report indicated that 58% of female and 59% of male adolescents who were never married reported to have used a condom during their last sexual practice [11]. The findings of this study are also corroborated with those of other studies, which revealed that despite knowing their HIV sero-status, HIV-positive adolescents still engage in risky behaviours [7, 16–18].

A good understanding of the transmission and prevention of STIs is crucial in HIV prevention in the population. Although this population had high knowledge of STI symptoms and complications, knowledge of STI transmission and prevention was significantly low. Previous studies in the country and elsewhere support these findings. Low comprehensive knowledge of STIs has been linked with low-risk perception, the practice of risky sexual behaviours and higher rates of HIV infection [11, 18–20]. The high knowledge of STI symptoms and complications found could be explained by the study being conducted in a health facility where some participants may have been seeking treatment for STI. The observed lack of important transmission and prevention knowledge among these young people indicates a gap in prevention among young people in the country. HIV prevention with a positive approach, which mainly dwells on the notion that ART treatment reduces viral load and, hence, HIV transmission, should be restructured to involve other dimensions such as knowledge and behavioural change. For an effective HIV prevention among HIV-positive young people, care and treatment centres should seize the opportunity of offering HIV transmission and prevention knowledge to clients.

Family support was found to play a significant role in reducing the practice of multiple sexual partnerships and secondary abstinence following HIV diagnosis. The psycho-social support provided by family members to young people living with HIV could be an opportunity for prevention of further HIV transmission [21].

In this study, participants who were on ART were significantly less likely to engage in the practice of multiple sexual partnerships as compared to those who were not on ART. Not being on ART may be associated with better health status and being able to engage in sexual activity [22]. Similarly, not being on ART gives a perception of good health status among people living with HIV/AIDS. Substance and alcohol consumption are believed to result in sexual dis-inhibition and impaired judgements, that is, multiple and unprotected sexual practices [18, 23, 24]. Regression analysis of independent factors for unprotected sex affirmed the observation of less use of condoms among alcohol users. The relatively lower practice of unprotected sex and less multiple sexual partnerships among those on ART may partly be explained by the observed low use of alcohol and the nature of their health status [25, 26]. Additionally, frequent visits for drug refills among those on treatment may have exposed them to frequent counselling and health education on prevention.

The majority of young people reported engaging in risk-sexual behaviours with partners of unknown HIV status. Lack of knowledge on the HIV status of the sexual partner was associated with a three times higher likelihood of reporting multiple sexual partners in this population [27]. The lower coverage of HIV counselling and testing in the country provides an opportunity for continued practice of multiple sexual partnership and HIV transmission as more adolescents are of unknown HIV status [11, 24]. Moreover, stigma associated with HIV/AIDS is a barrier to HIV disclosure programmes in the Country. More efforts to increase HIV testing and disclosure are needed to prevent the further spread of HIV infection and give people an opportunity to seek early treatment and care.

The findings of this study should be interpreted in light of the following limitations. First, the participants were recruited from health facilities and this to some extent limits the external validity of the results presented. Second, the study is based on self-reported information, which could be subject to desirability bias especially sexual behaviour-related information. Females are more likely to underreport and males overreport their sexual activities and this may have affected the magnitude of sexual behaviours and the associations reported. However, research assistants were trained and had counselling skills and conducted the interviews in private to ensure confidentiality. Third, due to an inherent weakness of the cross-sectional nature of the study, it is difficult to conclude whether the observed determinants of risky behaviours are causal. However, these determinants have been described as possible causal factors in analytical designs. Fourth, the knowledge scale categorization was based on scale distribution, which may not have theoretical grounds and this may result in a loss of information. The scale distribution observed seems to reflect this population knowledge characteristic based on knowledge distribution as reported in the demographic and health survey. Finally, it is important to acknowledge the potential for recall bias associated with self-reported behaviours. Participants might have forgotten some behaviours such a condom use and the number of sexual partners encountered.

## Conclusions

Unprotected sex and multiple sexual partnerships are prevalent among young people living with HIV in Dar Es Salaam. This heightens the concern on the risk among these young people of transmitting HIV infection to sero-discordant partners and re-infecting themselves with new-drug-resistant HIV strains. Practice of risky behaviours appears to be determined by location, age, alcohol consumption, family support, ART status and not knowing their sexual partner's HIV status.

Minimal knowledge on various aspects of STIs threatens risk perceptions facilitating the practice of risky behaviours and consequently leading to HIV transmission. Prevention with a positive approach incorporating preventive messages in HIV care and treatment is called for. These programmes should address the gap in STI knowledge, target substance and alcohol use and the practice of risky sexual behaviours. HIV testing campaigns that target young people are needed to improve HIV testing and disclosure as this may be of value in prevention and care.
